# Association between perceived neighbourhood characteristics, physical activity and diet quality: results of the Brazilian Longitudinal Study of Adult Health (ELSA-Brasil)

**DOI:** 10.1186/s12889-016-3447-5

**Published:** 2016-08-09

**Authors:** Dóra Chor, Letícia Oliveira Cardoso, Aline Araújo Nobre, Rosane Härter Griep, Maria de Jesus Mendes Fonseca, Luana Giatti, Isabela Bensenor, Maria del Carmen Bisi Molina, Estela M. L. Aquino, Ana Diez-Roux, Débora de Pina Castiglione, Simone M. Santos

**Affiliations:** 1 Escola Nacional de Saúde Pública, Fundação Oswaldo Cruz, Rio de Janeiro, RJ Brazil; 2 Programa de Computação Científica, Fundação Oswaldo Cruz, Rio de Janeiro, RJ Brazil; 3 Instituto Oswaldo Cruz, Fundação Oswaldo Cruz, Rio de Janeiro, 21040–360 RJ Brazil; 4 Escola de Nutrição, Universidade Federal de Ouro Preto, Ouro Preto, MG Brazil; 5 Hospital Universitário, Universidade de São Paulo, São Paulo, SP Brazil; 6 Departamento de Nutrição, Universidade Federal do Espírito Santo, Vitória, Espírito Santo Brazil; 7 Instituto de Saúde Coletiva, Universidade Federal da Bahia, Salvador, Bahia Brazil; 8 School of Public Health, Drexel University, Philadelphia, PA USA; 9 Fundação Oswaldo Cruz, Rio de Janeiro, RJ Brazil

**Keywords:** Neighbourhood, Physical activity, Diet, Food environment, Public health

## Abstract

**Background:**

The study explores associations between perceived neighbourhood characteristics, physical activity and diet quality, which in Latin America and Brazil have been scarcely studied and with inconsistent results.

**Methods:**

We conducted a cross-sectional analysis of 14,749 individuals who participated in the Brazilian Longitudinal Study of Adult Health (*Estudo Longitudinal de Saúde do Adulto*, ELSA-Brasil) baseline. The study included current and retired civil servants, aged between 35 and 74 years, from universities and research institutes in six Brazilian states. The International Physical Activity Questionnaire (IPAQ) long form was used to characterize physical activity during leisure time and commuting; additional questions assessed how often fruit and vegetables were consumed, as a proxy for diet quality. Neighbourhood characteristics were evaluated by the “Walking Environment” and “Availability of Healthy Foods” scales originally used in the *Multi-Ethnic Study of Atherosclerosis* (MESA). Associations were examined using multinomial logistic regression.

**Results:**

Perceiving a more walkable neighbourhood was positively associated with engaging in leisure time physical activity and doing so for longer weekly. Compared with those who saw their neighbourhood as less walkable, those who perceived it as more walkable had 1.69 (95 % CI 1.57–1.83) and 1.39 (1.28–1.52) greater odds of engaging in leisure time physical activity for more than 150 min/week or up to 150 min/week (vs. none), respectively. Perceiving a more walkable neighbourhood was also positively associated with transport-related physical activity. The same pattern was observed for diet: compared with participants who perceived healthy foods as less available in their neighbourhood, those who saw them as more available had odds 1.48 greater (1.31–1.66) of eating fruits, and 1.47 greater (1.30–1.66) of eating vegetables, more than once per day.

**Conclusions:**

Perceived walkability and neighbourhood availability of healthy food were independently associated with the practice of physical activity and diet quality, respectively, underlining the importance of neighbourhood-level public policies to changing and maintaining health-related habits.

**Electronic supplementary material:**

The online version of this article (doi:10.1186/s12889-016-3447-5) contains supplementary material, which is available to authorized users.

## Background

In Brazil’s state capitals, only 34 % of the adult population engage in open-air physical activity for the recommended length of time (≥150 min per week), while only 24 % consume at least five portions of fruit and vegetables on five or more days per week, one of the indicators of a healthy diet [1]. Physical activity is an important determinant of health and body weight, and is associated with the incidence of diabetes and cardiovascular diseases [2, 3]. In older adults, there is evidence that physical activity postpones functional decline [4] and dementia [5]. Unhealthy diets are associated with major public health problems, such as obesity, cardiovascular diseases and diabetes, both in Brazil and worldwide [6, 7].

Abel & Frohlich [8] posit that life chances (structure) and choice-based life conduct (agency) are jointly involved in shaping health-related choices and that the neighbourhood can be a place that fosters behavioural (or attitudinal) change. Investigations into how residential environment or neighbourhood characteristics influence health are important in generating empirical evidence to inform public policies based on the rationale that choice of health habits is not solely the individual’s responsibility, but is strongly related to constraints imposed by lack of material resources and by specific social groups’ behavioural norms [9, 10].

In recent decades, several epidemiological studies have described the effects of neighbourhood characteristics on health-related behaviour that affect morbidity and mortality [11–14]. Although the specific mechanisms that may explain the effect of neighbourhood on health-related behaviour are not yet clearly established, there is evidence that easier access to healthy diet and neighbourhoods that offer conditions conducive to engaging in physical activity do increase the likelihood of healthy habits [11, 15–17].

One of the major challenges in studying the effects of neighbourhood on patterns of health-related behaviour, including physical activity and diet quality, is to identify what characteristics may be associated with such behaviour. Given that this literature is still sparse in middle-income countries, such as Brazil, it is important to evaluate a range of characteristics – e.g., traffic, the presence of people exercising –by means of an instrument used previously in another context, the *Multi-Ethnic Study of Atherosclerosis* (MESA) [18, 19]. In addition, objective and/or perceived measures of neighbourhood characteristics have been used, with both presenting advantages and limitations [19–22]. For example, the measurement of types of food stores present in the areas in which participants live may be an important and useful tool for describing the food environment. However, important dimensions of access that are key factors in environment-diet relationship such as affordability, food choice and acceptability can only be assessed by participants report. Similarly, actual crime rates appear to be a worse predictor of physical inactivity than fear of crime. A limitation of perceived measures, on the other hand, is the possibility of reporting bias generated by reports of neighbourhood conditions and self-reported outcomes. In this study, we use the study population’s perception of their neighbourhood’s characteristics, because these can “operate as key pathway linking objective neighbourhood material circumstances to health” [20].

Previous studies of the association between neighbourhood characteristics and physical activity and consumption of healthy foods have yielded inconsistent results. We performed a cross-sectional analysis of the relation between perceived neighbourhood characteristics and these two types of behaviour in the baseline results of the Longitudinal Study of Adult Health (*Estudo Longitudinal de Saúde do Adulto*, ELSA-Brasil), a multicentre cohort study to investigate the development of chronic diseases, particularly cardiovascular disease and diabetes. Our hypotheses were: 1) participants who perceive characteristics that favour physical activity in their neighbourhood engage in more leisure-time, and to a lesser extent transport-related, physical activity; and 2) participants who perceive greater healthy food availability in their neighbourhood consume more fruit and vegetables.

## Methods

### Study participants

The details of the ELSA-Brasil – including design, eligibility criteria, sources and methods of recruitment and participant characteristics – have been described in detail elsewhere [23, 24]. Briefly, it is a multicentre cohort study designed to estimate the incidence of cardiovascular diseases and diabetes, as well as their main social, environmental, occupational and biological determinants. It involves civil servants at teaching and research institutions in six Brazilian cities (Rio de Janeiro, São Paulo, Vitória, Belo Horizonte, Bahia and Porto Alegre). All active or retired employees of the six institutions aged 35 to 74 years were eligible for the study. Baseline assessment took place from August 2008 to December 2010 and consisted of an approximately 7-hour evaluation, which included in-person interviews conducted by trained personnel and a comprehensive set of examinations and measurements such as transthoracic echocardiography, carotid artery intima-media thickness and retinal fundus photography besides the more usual ones as oral glucose tolerance test, anthropometry and blood pressure measurement [25, 26]. The study was approved by the Research and Ethics Committees of the institutions involved - Oswaldo Cruz Foundation and the federal universities of Bahia, Espirito Santo, Minas Gerais, and Rio Grande do Sul; and the University of Sao Paulo. All participants signed declarations of informed consent.

For the baseline of this cohort study, all active or retired employees of the six institutions aged 35–74 years were eligible for the study, totalling, in 2008, 52 137 potential participants. As a cohort study (which is now beginning its wave three of data collection), we estimated a sample size which would be sufficient to its main objectives. Sample size estimation was based on the main study outcomes—type 2 diabetes and myocardial infarction (cardiovascular disease). In order to present gender-specific analyses and allow for possible losses to follow-up, we defined the desired sample size as approximately 15,000 persons. Efforts were made to recruit similar proportions of men and women as well as predefined proportions of age groups and occupational categories. From a total of 16 435 who expressed interest in participation, 15 821 were pre-enrolled, gave written consent, responded to an initial interview and were scheduled for the baseline examination. Only 716 (4.5 %) of those pre-enrolled did not complete the baseline examination. A total of 15,105 participants were enrolled; this study comprised 14,749 participants with complete data on all variables of interest.

### Data collection

#### Exposure assessment

The scales used to evaluate neighbourhood characteristics – originally used in the *Multi-Ethnic Study of Atherosclerosis* (MESA) [18] – were adapted to Brazilian Portuguese in connection with the ELSA-Brasil study [27]. These instruments record respondents’ perceptions with regard to social and physical characteristics of their neighbourhood environment. Interviewees were asked to think about their neighbourhood as follows: “By neighbourhood we mean the area around where you live and around your house. It may include places you shop, religious or public institutions, or the local business district. It is the general area around your house where you might perform routine tasks, such as shopping, going to the park, or visiting neighbours”.

The two scales applied to these analyses were: 1) Walking Environment (nine items) and 2) Availability of Healthy Foods (four items) (Additional file 1). Response values ranged from 1 to 5 (agree completely to disagree completely). In the Brazilian context [27] and in international studies [28, 29], these scales displayed appropriate psychometric properties with Cronbach’s alpha’s ranging from 0.60 to 0.94 and with test-retest correlations ranging from 0.78 to 0.91.

On the scale evaluating walkability in ELSA-Brasil, scores varied from 9 (perception of better quality) to 45 (perception of worse quality). Based on the distribution of the responses to each item of the scale, a cut-off score of 18 was applied to divide the two groups: perception of “better walkability” (score ≤ 18) and “worse walkability” (score > 18). That cut-off point has been chosen because it was considered the best to separate two different groups: scores of less than 18 mean that most of the responses for that scale varied between ‘agree completely’ and ‘agree partly’, indicating the ‘better walkability’ group, whereas scores of 18 or more concentrated most of the responses that indicated ‘worse walkability’. The same criterion was applied to the scale evaluating neighbourhood availability of healthy foods, with scores varying between 4 (perception of better quality) and 20 (perception of worse quality). The cut-off score was set at 8: “better availability” (score ≤ 8) and “worse availability” (score >8).

#### Outcome assessment

In the ELSA-Brasil study, the International Physical Activity Questionnaire (IPAQ) long form was used to assess physical activity during leisure time and for transport [30, 31]. The questions about leisure time physical activity cover the weekly frequency and duration of walking and physical activities of moderate or vigorous intensity performed for at least ten minutes at a time. Transport-related questions include frequency and duration of on foot or by bicycle physical activity. For analytical purposes, an approximate measure of the ‘regularity’ of physical activity was obtained by multiplying the number of days on which physical activity was practised by the duration in minutes. Participants were then classified into three frequency levels: 1) no physical activity; 2) <150 min/week; and 3) ≥ 150 min/week. This cut-off point was chosen for having been recommended by leading institutions in adult health promotion and maintenance [32–34].

Individual consumption of fruit and vegetables was used as a proxy for diet quality and was measured by way of two questions: ‘How often do you usually consume fruit?’ and ‘How often do you eat raw, boiled or sautéed vegetables, excluding potatoes, cassava and yam?’ Since 2006, those questions have been routinely used by ‘VIGITEL’, the Brazilian Risk Factor Surveillance for Non-Transmissible Chronic Diseases by Telephone Survey with good psychometric properties [35]. There were eight response options: 1) More than three times a day; 2) Two or three times a day; 3) Once a day; 4) Five to six times a week; 5) Two to four times a week; 6) Once a week; 7) One to three times a month; and 8) Never or almost never. For both questions, responses were grouped into frequency categories: high (twice or more per day); daily (once a day/five to six times a week); weekly (two to four times a week); and rarely (once a week or less).

#### Covariable assessment

The covariables considered were: gender, age, education (<elementary, elementary complete, secondary complete, university graduate, postgraduate) and monthly per capita family income, calculated from the midpoint of the reported net income category divided by the number of people dependent on that income. Given the wide variation in this variable, it was standardised by subtracting the mean value and dividing by the standard deviation. Per capita family income was converted into US dollars using the 2009 Purchasing Power Parity (PPP) conversion factor for private consumption (BRL 1.7 = USD 1), as published annually by the World Bank [36]. Length of time residing in the neighbourhood was measured in years.

### Statistical analysis

Multinomial logistic regression analysis was used to examine the cross-sectional association between the exposure variables (perception of the neighbourhood in terms of walkability and availability of healthy foods) and the outcomes of interest (physical activity and diet). Crude and adjusted associations were computed (model 1: crude; model 2: adjusted for age; model 3: adjusted for age and gender; model 4: adjusted for age, gender and education; model 4: adjusted for age, gender education and standardised income score. Interaction between gender and the exposure variables was tested. The analyses were performed on the *nnet* library of R version 2.15 [37].

## Results

Of the participants, 8023 (54 %) were women, 7787 (53 %) had a university degree, median age was 51 years and mean time of residence in the neighbourhood was 17 years (Table 1). Mean monthly per capita family income was BRL 1751.00, corresponding, on Purchasing Power Parity (PPP), to USD 1029.95. Perception of worse walkability was reported more frequently by women (Table 1), by the group with least mean per capita income and varied inversely with education. Thus, 564 (65 %) of those who had not completed elementary education perceived worse walkability, as compared with 2514 (46 %) of the postgraduate group. In addition, the group that perceived worse walkability showed lower median age and longer time of residence in the neighbourhood. During leisure time, women, individuals with lower levels of education and lower mean per capita income were the least active or, when they did engage in physical activity, did so for the least time weekly. As for transport, 28 and 24 % of women and men, respectively, reported no physical activity (Table 1). Among those who did engage in transport-related physical activity, most walked and only 5.8 % rode a bicycle (results not shown). Of note, those with higher levels of education and higher mean per capita income were less active than their counterparts with less favourable socioeconomic conditions.

**Table 1:** Perceived walkability and weekly physical activity by population characteristics – ELSA-Brasil, 2008–2010 (*n* = 14,749)

Characteristics	n	Perceived walkability		Leisure-time physical activity/week			Transport-related physical activity/week		
		Better	Worse	No	<150 min	> = 150 min	No	<150 min	> = 150 min
Gender									
Women	8023	42.8	57.2	48.0	20.5	31.4	27.7	39.6	32.6
Men	6726	44.9	55.1	37.0	23.1	40.0	24.4	39.2	36.5
Education									
< Elementary	869	35.1	64.9	57.9	19.7	22.4	17.3	40.4	42.3
Elementary	1002	35.3	64.7	53.3	20.7	26.0	19.1	40.9	40.0
Secondary	5091	36.1	63.9	52.0	19.5	28.5	21.5	36.8	41.6
University graduate	2345	43.8	56.2	30.5	24.4	45.1	32.4	41.6	26.0
Postgraduate	5442	53.8	46.2	42.6	21.3	36.1	28.3	39.0	32.7
Median age (years)	51.0	52.0	51.0	51.0	52.0	52.0	51.0	51.0	52.0
Mean per capita income (R$)	1751.00	1984.71	1569.44	1431.50	1857.57	2074.45	1958.87	1783.45	1555.48
Mean years residence in the neighbourhood	17.2	15.9	18.3	17.7	16.7	16.9	16.3	16.8	18.3

A less consistent pattern was observed for perceived availability of healthy food in the neighbourhood (Table 2). Education was not consistently associated with perceived availability of healthy foods: in the group with less than complete elementary education, 363 (41.8 %) of participants perceived worse availability of healthy food, compared to 1088 (46.4 %) in university graduates and 2184 (40.1 %) in postgraduates. Men, the group with least education and those with less mean per capita income consumed fruit and vegetables less frequently.

**Table 2:** Perceived availability of healthy food and fruit and vegetable consumption by population characteristics – ELSA-Brasil, 2008–2010

Characteristics	n	Perceived availability of healthy food		Frequency of fruit consumption				Frequency of vegetable consumption			
		Better	Worse	> Once/day	Daily	Weekly	Rare	> Once/day	Daily	Weekly	Rare
Gender											
Female	8023	56.7	43.3	27.9	45.3	17.6	9.2	16.9	51.3	20.9	10.9
Male	6726	56.2	43.8	16.1	40.3	25.7	17.9	12.2	44.0	25.9	17.9
Education											
< Elementary	869	58.2	41.8	19.8	36.7	21.4	22.1	14.2	32.6	29.5	23.7
Elementary	1002	55.8	44.2	18.4	38.2	23.8	19.6	13.5	35.6	28.1	22.8
Secondary	5091	54.0	46.0	18.7	42.4	23.0	16.0	13.2	42.7	26.0	18.1
University graduate	2345	53.6	46.4	20.4	45.5	22.2	11.9	13.5	49.3	24.6	12.5
Postgraduate	5442	59.9	40.1	28.3	44.5	18.7	8.5	17.1	57.0	18.0	7.9
Median age (years)	51.0	52.0	50.0	54.0	52.0	49.0	50.0	52.0	52.0	51.0	50.0
Mean per capita income (R$)	1751.00	1847.10	1626.22	2071.99	1808.04	1529.64	1371.99	1856.81	1957.76	1535.36	1290.80
Mean years residence in the neighbourhood	17.2	17.5	16.8	18.1	17.2	16.3	17.2	17.6	17.0	17.1	17.9

Perceived walkability showed a positive association both with engaging in leisure time physical activity and with its weekly duration (Table 3). Among those who perceived better walkability*,* the odds of engaging in physical activity for longer than 150 min/week (vs. not engaging in leisure time physical activity) were 1.69 times greater (95 % confidence interval (CI)1.57–1.83) than among those who perceived worse walkability. The odds of engaging in physical activity for up to 150 min/week (vs. doing none at all) were 1.40 greater (95 % CI 1.28–1.52) among those who perceived better walkability. The interaction term between walkability and gender was not statistically significant (*p* = 0.13). Perceived walkability was positively associated with higher duration of physical activity for transport (>150 min/week). Among those who perceived better walkability*,* the odds of engaging in physical activity for longer than 150 min/week (vs. not engaging in transport-related physical activity) were 1.19 times greater (95 % CI 1.09–1.30) than among those who perceived worse walkability.

**Table 3:** Association between perceived walkability and physical activity – ELSA-Brasil, 2008–2010

Variables	Leisure-time physical activity				Transport-related physical activity			
	<150		> = 150		<150		> = 150	
	OR	CI(95 %)	OR	CI(95 %)	OR	CI(95 %)	OR	CI(95 %)
Model 1								
Walkability (better)	**1.57**	**1.44 – 1.71**	**1.98**	**1.84 – 2.14**	1.02	0.94 – 1.11	1.03	0.95 – 1.12
Model 2: Model 1 + age								
Walkability (better)	**1.56**	**1.43 – 1.70**	**1.97**	**1.83 – 2.12**	1.01	0.93 – 1.10	1.02	0.94 – 1.11
Age	1.01	1.00 – 1.01	**1.02**	**1.01 – 1.02**	1.01	1.00 – 1.01	**1.02**	**1.01 – 1.02**
Model 3: Model 2 + gender								
Walkability (better)	**1.56**	**1.43 – 1.70**	**1.97**	**1.82 – 2.12**	1.01	0.93 – 1.10	1.01	0.93 – 1.10
Age	1.01	1.00 – 1.01	**1.02**	**1.01 – 1.02**	1.01	1.00 – 1.01	**1.02**	**1.01 – 1.02**
Gender (male)	**1.46**	**1.34 – 1.59**	**1.65**	**1.53 – 1.77**	**1.12**	**1.04 – 1.22**	**1.27**	**1.17 – 1.38**
Model 4: Model 3 + education								
Walkability (better)	**1.42**	**1.30 – 1.55**	**1.73**	**1.60 – 1.87**	1.07	0.99 – 1.17	**1.17**	**1.08 – 1.28**
Age	**1.01**	**1.01 – 1.02**	**1.02**	**1.02 – 1.02**	1.01	1.00 – 1.01	**1.02**	**1.01 – 1.02**
Gender (male)	**1.51**	**1.39 – 1.65**	**1.75**	**1.62 – 1.90**	**1.11**	**1.02 – 1.20**	**1.27**	**1.16 – 1.38**
Education (Elementary complete)	1.21	0.96 – 1.54	**1.39**	**1.11 – 1.74**	0.94	0.73 – 1.22	0.91	0.70 – 1.18
(Secondary complete)	**1.31**	**1.08 – 1.59**	**1.83**	**1.52 – 2.20**	**0.79**	**0.64 – 0.98**	0.94	0.76 – 1.16
(University graduate)	**2.59**	**2.14 – 3.15**	**4.37**	**3.64 – 5.24**	**0.57**	**0.47 – 0.70**	**0.36**	**0.29 – 0.44**
(Postgraduate)	**1.78**	**1.44 – 2.20**	**2.89**	**2.37 – 3.51**	**0.64**	**0.51 – 0.80**	**0.56**	**0.45 – 0.70**
Model 5: Model 4 + income								
Walkability (better)	**1.40**	**1.28 – 1.52**	**1.69**	**1.57 – 1.83**	1.08	0.99 – 1.17	**1.19**	**1.09 – 1.30**
Age	1.01	1.00 – 1.01	**1.01**	**1.01 – 1.02**	1.01	1.00 – 1.01	**1.02**	**1.02 – 1.03**
Gender (male)	**1.53**	**1.41 – 1.68**	**1.80**	**1.66 – 1.94**	**1.10**	**1.01 – 1.20**	**1.25**	**1.15 – 1.36**
Education (Elementary complete)	1.18	0.93 – 1.50	**1.34**	**1.07 – 1.67**	0.95	0.74 – 1.23	0.93	0.72 – 1.20
(Secondary complete)	**1.22**	**1.01 – 1.48**	**1.65**	**1.37 – 1.98**	0.81	0.66 – 1.00	0.99	0.80 – 1.22
(University graduate)	**1.98**	**1.61 – 2.44**	**2.98**	**2.46 – 3.62**	**0.63**	**0.51 – 0.78**	**0.44**	**0.35 – 0.55**
(Postgraduate)	**1.51**	**1.22 – 1.87**	**2.28**	**1.86 – 2.79**	**0.68**	**0.54 – 0.85**	**0.64**	**0.51 – 0.80**
Per capita income z-score	**1.22**	**1.15 – 1.29**	**1.32**	**1.25 – 1.38**	**0.94**	**0.90 – 0.99**	**0.87**	**0.83 – 0.92**

Reference category = no physical activity

Bold data are statistically significative

The pattern of association between perceived availability of healthy food in the neighbourhood and frequency of consuming fruit and vegetables was similar to that observed for the physical activity variables. Compared with the participants who perceived worse availability, those who perceived better availability of healthy foods had 1.48 times greater odds (95 % CI 1.32–1.66) of consuming fruit more than once a day (vs. rarely) (Table 4). Those who perceived better availability returned adjusted odds 1.47 times greater (95 % CI 1.30-1.67) of consuming vegetables more than once a day (vs. rarely) (Table 5). The interaction term between gender and perceived availability of healthy food was not significant (*p* = 0.43 and *p* = 0.52 for fruit and vegetables, respectively).

**Table 4:** Association between perceived availability of healthy foods and frequency of consumption of fruit – ELSA-Brasil, 2008–2010

	Consumption of fruit^a^					
Variables	Weekly		Daily		High	
	OR	CI(95 %)	OR	CI(95 %)	OR	CI(95 %)
Model 1						
Neighbourhood availability of healthy foods (better)	**1.01**	**0.91 – 1.14**	**1.37**	**1.24 – 1.52**	**1.65**	**1.47 – 1.84**
Model 2: Model 1 + age						
Neighbourhood availability of healthy foods (better)	**1.02**	**0.91 – 1.14**	**1.31**	**1.18 – 1.45**	**1.53**	**1.36 – 1.71**
Age	1	0.99 – 1.00	1.03	1.02 – 1.04	1.05	1.04 – 1.05
Model 3: Model 2 + gender						
Neighbourhood availability of healthy foods (better)	**1.02**	**0.91 – 1.15**	**1.32**	**1.19 – 1.46**	**1.55**	**1.38 – 1.74**
Age	1	0.99 – 1.00	1.03	1.02 – 1.04	1.05	1.04 – 1.06
Gender (male)	0.76	0.67 – 0.85	0.45	0.4 – 0.5	0.28	0.25 – 0.32
Model 4: Model 3 + education						
Neighbourhood availability of healthy foods (better)	**1.02**	**0.91 – 1.14**	**1.30**	**1.17 – 1.44**	**1.50**	**1.33 – 1.68**
Age	1.00	0.99 – 1.01	1.04	1.03 – 1.04	1.06	1.05 – 1.06
Gender (male)	0.77	0.69 – 0.87	0.46	0.42 – 0.51	0.28	0.25 – 0.32
Education (Elementary complete)	1.24	0.94 – 1.63	1.23	0.96 – 1.58	1.11	0.82 – 1.49
(Secondary complete)	1.42	1.13 – 1.78	1.82	1.48 – 2.23	1.56	1.23 – 1.99
(University graduate)	1.8	1.4 – 2.33	2.5	1.98 – 3.15	2.11	1.61 – 2.75
(Postgraduate)	2.21	1.75 – 2.80	3.56	2.88 – 4.41	4.41	3.47 – 5.62
Model 5: Model 4 + standardised income score						
Neighbourhood availability of healthy foods (better)	**1.02**	**0.91 – 1.14**	**1.29**	**1.16 – 1.43**	**1.48**	**1.32 – 1.66**
Age	1	0.99 – 1.01	1.03	1.03 – 1.04	1.05	1.04 – 1.06
Gender (male)	0.77	0.69 – 0.87	0.46	0.42 – 0.52	0.29	0.25 – 0.32
Education (Elementary complete)	1.24	0.94 – 1.64	1.21	0.94 – 1.56	1.08	0.8 – 1.45
(Secondary complete)	1.42	1.13 – 1.78	1.75	1.42 – 2.15	1.46	1.15 – 1.87
(University graduate)	1.81	1.39 – 2.36	2.27	1.78 – 2.89	1.8	1.36 – 2.37
(Postgraduate)	2.23	1.72 – 2.89	3.06	2.42 – 3.86	3.42	2.62 – 4.46
Per capita income z-score	1	0.91 – 1.08	1.12	1.04 – 1.21	1.2	1.10 – 1.30

Reference category: rarely consumed (once a week or less). ^a^weekly (two to four times a week); daily (once a day/five to six times a week); high (twice or more a day)

Bold data are statistically significative

**Table 5:** Association between perceived availability of healthy food and frequency of consumption of vegetables – ELSA-Brasil, 2008–2010

Variables	Consumption of vegetables^a^					
	Weekly		Daily		High	
	OR	CI(95 %)	OR	CI(95 %)	OR	CI(95 %)
Model 1						
Neighbourhood availability of healthy foods (better)	**1.11**	**0.99 – 1.23**	**1.37**	**1.25 – 1.52**	**1.57**	**1.39 – 1.77**
Model 2: Model 1 + age						
Neighbourhood availability of healthy foods (better)	**1.10**	**0.98 – 1.22**	**1.34**	**1.21 – 1.48**	**1.53**	**1.35 – 1.73**
Age	1	1 – 1.01	1.01	1.01 – 1.02	1.02	1.01 – 1.02
Model 3: Model 2 + gender						
Neighbourhood availability of healthy foods (better)	**1.10**	**0.98 – 1.22**	**1.34**	**1.21 – 1.48**	**1.53**	**1.35 – 1.73**
Age	1	1 – 1.01	1.02	1.01 – 1.02	1.02	1.01 – 1.02
Gender (male)	0.76	0.68 – 0.85	0.52	0.47 – 0.58	0.44	0.39 – 0.5
Model 4: Model 3 + education						
Neighbourhood availability of healthy foods (better)	**1.09**	**0.98 – 1.22**	**1.3**	**1.17 – 1.44**	**1.49**	**1.31 – 1.68**
Age	1.01	1 – 1.01	1.02	1.01 – 1.03	1.02	1.01 – 1.03
Gender (male)	0.77	0.69 – 0.86	0.53	0.48 – 0.59	0.44	0.39 – 0.5
Education (Elementary complete)	1	0.78 – 1.29	1.16	0.9 – 1.48	0.99	0.72 – 1.35
(Secondary complete)	1.16	0.94 – 1.43	1.78	1.45 – 2.18	1.21	0.94 – 1.56
(University graduate)	1.57	1.23 – 1.99	2.85	2.27 – 3.58	1.7	1.28 – 2.26
(Postgraduate)	1.85	1.49 – 2.31	5.45	4.41 – 6.73	3.65	2.82 – 4.72
Model 5: Model 4 + standardised income score						
Neighbourhood availability of healthy foods (better)	**1.08**	**0.97 – 1.21**	**1.28**	**1.16 – 1.42**	**1.47**	**1.30 – 1.67**
Age	1.01	1 – 1.01	1.01	1.01 – 1.02	1.02	1.01 – 1.02
Gender (male)	0.78	0.69 – 0.87	0.54	0.49 – 0.6	0.44	0.39 – 0.5
Education (Elementary complete)	0.99	0.77 – 1.27	1.12	0.87 – 1.43	0.96	0.7 – 1.32
(Secondary complete)	1.12	0.9 – 1.38	1.62	1.32 – 1.99	1.13	0.88 – 1.46
(University graduate)	1.41	1.1 – 1.81	2.29	1.81 – 2.91	1.45	1.08 – 1.94
(Postgraduate)	1.56	1.22 – 2	3.83	3.04 – 4.84	2.81	2.12 – 3.73
Per capita income z-score	1.14	1.05 – 1.25	1.30	1.21 – 1.41	1.23	1.12 – 1.34

Reference category: rarely consumed (once a week or less). ^a^weekly (two to four times a week); daily (once a day/five to six times a week); high (twice or more a day)

Bold data are statistically significative

## Discussion

This study of civil servants living in six different cities in Brazil found that perceiving the environment as more favourable to physical activity was associated with leisure-time physical activity (and with that activity being practised for longer weekly durations) and with transport-related physical activity. In the same direction, the perception of better availability of healthy food in the neighbourhood displayed an association with healthier diet represented by greater consumption of fruit and vegetables.

The multiplicity of instruments for measuring neighbourhood characteristics limits comparison among studies and may partly explain reports of lack of association between neighbourhood characteristics and physical activities [38, 39]. In addition, some authors have investigated specific aspects, such as how levels of violence are associated with people’s leisure time and transport-related physical activities [40, 41]. In our case, the scale comprises various conditions that may affect the practice of physical activity, such as the presence of clubs, squares, street conditions, heavy traffic and trees [18, 27, 29]. Taking those differences into consideration, our results confirm those reported in compilations of studies in different countries [40, 42, 43] that a positive relation exists between neighbourhood characteristics and physical activities. With regard to leisure time physical activity, additional evidence presented in a population-based study in Seoul, South Korea, showed that satisfaction with security and with the existence of free park and recreation facilities in the neighbourhood was positively associated with vigorous physical activity among women [44]. In the United States, an environment perceived as favourable in terms of aesthetics, street quality, traffic safety and low levels of violence had a positive association on use of public spaces and facilities for recreation and physical activity [45, 46]. In Holland, Jongeneel-Grimen et al. [47, 48] presented strong evidence of a causal relation between neighbourhood-related factors and the practice of physical activity. In Curitiba – a city in Southern Brazil, whose human development index (HDI) is among the country’s ten highest [49] – characteristics such as the “existence of interesting things to see in the neighbourhood”, “existence of trees in the neighbourhood”, “number of positive aesthetic and security attributes in the neighbourhood” showed a positive association with use of public spaces for recreation and leisure [50].

We found that perceptions of the environment as more favourable to physical activity were associated with transport-related physical activity, although the association was weaker than with leisure time physical activity. Also, the association existed only for the longest duration of activity (>150 min/week). Most authors [40, 51, 52], but not all [53], have reported similarly positive associations. In 17 cities (12 countries), Kerr et al. [40] investigated perceived environmental attributes such as residential density, land use mix-access (having easy access to shops, recreational spaces) and traffic and crime safety, which were found to be associated with transport-related physical activity. The results of studies conducted in four Brazilian cities have also shown association, which vary in direction by specific characteristic [54–57]. Our results confirm the existence of the association and suggest that people who perceive better walkability may live in neighbourhoods offering conditions, such as large sidewalks, street connectivity and safer traffic, that encourage them to walk to and from bus, metro or train stop or, when possible, to walk straight to their destination. Large traffic jams have been a strong motivation for people to leave their cars at home and use public transport, where they can use their travel time to work, read or simply relax. On the other hand, participants with better perceived walkability and less advantageous socioeconomic conditions may walk for transport or mostly, to and from transport, because they live in areas not serviced by public transport, do not own a car or walking is their least expensive option.

In Brazil, the association of food environment on diet quality has been little studied. The results of an ecological study in São Paulo City indicated the density of stores specialising in selling fruit and vegetables was associated positively with regular consumption of such foods [58]. Our results agree with those of the MESA (ELSA-Brasil applied the same neighbourhood evaluation instrument), in which participants living in the areas ranked worst in food availability were 22–35 % less likely to have a healthy diet than those in the best-ranked areas [19]. A recent review of studies from various countries found most indicating that perceptions of greater availability, variety and affordability of foods associated directly with consumption of fruit and vegetables [59]. That review reported moderate evidence of a causal relationship between neighbourhood food environment and type of diet, given that some studies found no such association [60, 61].

Theoretical models able to elucidate the complex mechanisms that relate environment with healthy habits are still under construction [59, 62, 63]. For consumption to occur, material resources must be available when supply is not free (e.g., public spaces for practising physical activity) and foods are not accessibly priced. In addition to the positive associations between education, income and greater consumption of fruit and vegetables found in our study, a recent survey in São Paulo found that access to healthy foods was greater in areas of the city with medium and high socioeconomic status [64]. Another mechanism that may explain the relation between objective conditions that favour healthy habits and such habits actually being practised is the awareness that they are important and individual perceptions that these characteristics are present in the neighbourhood [16]. The availability of such structures in the neighbourhood makes for greater access and may kindle interest in changing habits relating to health and wellbeing. However, these relations are more complex. For instance, a study by Cummins et al. [65] in Glasgow, Scotland, showed that after a new supermarket was built in an area where there had been no such establishment, the local population’s consumption of fruit and vegetables showed no change, despite distribution of purchase vouchers. One of the explanations offered is that the population did not ‘identify or recognise themselves’ as consumers in that environment. Accordingly, availability seems to be mediated by values and cultural habits, as well as by diverse demographic and psychological characteristics [18].

It is worth noting that in countries like Brazil people who can afford to live in more expensive neighbourhoods also tend to have access to a series of facilities including greater availability of healthy foods and services, public and private spaces and more secure conditions for practising physical activity [64]. In our study population, environments perceived as better for engaging in physical activity and perceived better availability of healthy food were also more frequent among individuals with better education and higher per capita family income. However, the perception of neighbourhood showed a significant association with both behaviour types studied, even after adjustment for these characteristics, suggesting it is affected by context, as well as by individual socioeconomic status.

The strengths of our study are related primarily to the ELSA-Brasil itself, the first cohort study of cardiovascular diseases and diabetes in Latin America, which placed major emphasis on the social and contextual determinants of health. Given that most other studies have been conducted in high-income countries, our results contribute to understanding the associations of interest in different context. In addition, as it is multicentre, it is possible to derive results relating to some 15,000 individuals resident in six metropolises located in differing regions of Brazil (South, Southeast and Northeast). We also used a structured, validated questionnaire similar to the one applied in one of the main cohort studies of cardiovascular diseases [18], making it easier to compare results.

However, some potential limitations of our analysis merit consideration. Both cross-sectional and longitudinal analyses [66] may display migration bias from physically active individuals seeking to live in places favourable to such habits and the same occurring in relation to the availability of healthy food [47, 67]. We were unable to control for self-selection to the neighbourhood. However, it must be acknowledged that most people in Brazil do not choose where they live; to some extent, they are chosen (or excluded) by housing prices in the various different areas of the city. To some extent, that is a type of bias because “high-class” areas are also the ones that offer the best objective conditions for acquiring and maintaining healthy habits. It is thus unlikely that people made a priority of choosing their place of residence specifically with a view to healthy habits, especially some 17 years ago (our population’s mean time of residence in the neighbourhood), when the relation between healthy habits, health and wellbeing had not yet been popularised in Brazil. Similar to other cohort studies [68, 69], our sample does not represent the Brazilian population. Mean family income and education attainment in ELSA-Brasil are higher than in Brazilian population. However, there is enough variability regarding socioeconomic position, allowing for comparisons among groups. Finally, we cannot exclude same source bias (i.e. possibility that people with better diets are more aware of healthy foods, same for physical activity). Ongoing analyses considering aggregates across areas will further elucidate this possibility.

## Conclusions

Perceived walkability and neighbourhood availability of healthy food were independently associated with the practice of physical activity and diet quality, respectively. Those results reinforce evidence that developing healthy habits does not depend solely on the individual [8], underlining the importance of neighbourhood-level public policies to changing and maintaining health-related habits. In countries with considerable social inequality, public policies constitute one of the key means of creating objective conditions for embracing and maintaining such behaviour. How relations between residential context and behaviour develop over time will be addressed by future longitudinal analyses of the ELSA-Brasil cohort. In addition, in connection with that study, it will be possible to experiment by combining different approaches to evaluating context, including geographical location measurements, other indicators extracted from secondary data sets and individual perceptions, which are strategies that have been little used simultaneously in countries like Brazil.

## Abbreviations

ELSA, Estudo Longitudinal de Saúde do Adulto (*Longitudinal Study of Adult Health*); HDI, human development index; IPAQ, International Physical Activity Questionnaire; MESA*,* multi-ethnic study of atherosclerosis; PPP, purchasing power parity

## Additional file

Additional file 1: Items included in the neighbourhood scales. This additional file presents the items included in the neighbourhood scales, which report the participants’ perception of the walking environment and availability of healthy foods. (PDF 40 kb)
