# Supplementary material for: Association between perceived neighbourhood characteristics, physical activity and diet quality: results of the Brazilian Longitudinal Study of Adult Health (ELSA-Brasil)
Source: BMC Public Health. 2016 Aug 9;16:751. doi: 10.1186/s12889-016-3447-5 (PMC4977638; doi:10.1186/s12889-016-3447-5)
Supplement: Additional file 1: — Items included in the neighbourhood scales. This additional file presents the items included in the neighbourhood scales, which report the participants’ perception of the walking environment and availability of healthy foods. (PDF 40 kb) [file 12889_2016_3447_MOESM1_ESM.pdf]

Additional file 1

Items included in the neighbourhood scales

Walking Environment

1. My neighbourhood offers many opportunities to be physically active
2. Local sports clubs and other facilities in my neighbourhood offer many opportunities to get exercise
3. It is pleasant to walk in my neighbourhood
4. The trees in my neighbourhood provide enough shade
5. In my neighbourhood it is easy to walk places
6. I often see other people walking in my neighbourhood
7. I often see other people exercising (for example, jogging, bicycling, playing sports) in my neighbourhood
8. My neighbourhood has heavy traffic
9. There are many busy roads to cross when out for walks in my neighbourhood

Availability of Healthy Foods

1. A large selection of fresh fruits and vegetables is available in my neighbourhood
2. The fresh fruits and vegetables in my neighbourhood are of high quality
3. A large selection of low-fat products is available in my neighbourhood
4. There are many opportunities to purchase fast foods in my neighbourhood
